# From discarding to leveraging: quality-aware collaborative learning for robust diabetic retinopathy grading

**DOI:** 10.3389/fmed.2026.1795484

**Published:** 2026-05-29

**Authors:** Yuan Pan, Xiangwen Cai, Pan Xiong, Fang Mei, Zhengfei Wang, Jiaming Hong

**Affiliations:** 1 School of Medical Information Engineering, Guangzhou University of Chinese Medicine, Guangzhou, China; 2 Guangdong Province Key Laboratory of Popular High Performance Computers, College of Computer Science and Software Engineering, Shenzhen University, Shenzhen, China

**Keywords:** collaborative learning, diabetic retinopathy grading, dynamic expert routing, fundus image quality assessment, multi-scale feature learning

## Abstract

Real-world diabetic retinopathy (DR) screening faces a paradox: the most diagnostically critical images are often the lowest in quality, because advanced disease itself produces vitreous hemorrhage, proliferative tissue, and media opacities that degrade fundus imagery. We characterize this quality-severity coupling quantitatively (Spearman ρ = 0.420, odds ratio 4.17 for referable DR in Reject vs. Good strata on DDR, *p* < 0.001) and show that conventional pipelines work against it: filtering low-quality images discards the most severe cases, while uniform processing leads to misclassification. Both behaviors stem from treating image quality assessment (IQA) as a binary preprocessing decision. We argue that quality should serve as a continuous guidance signal that conditions the diagnostic process, and propose QGDR, a quality-guided dynamic routing framework realizing this paradigm through three coordinated mechanisms: (i) a multi-level IQA module that extracts hierarchical quality features across backbone stages; (ii) a quality-conditioned context gating mechanism that modulates spatial attention according to the predicted quality state; and (iii) an adaptive gated fusion mechanism that routes inputs to scale-specialized experts, with high-quality images preferentially activating fine-scale experts for subtle lesions and degraded images relying on coarse-scale experts for robust global pattern recognition. On EyeQ and DDR, QGDR attains 78.32% accuracy with 0.6863 QWK and 80.85% accuracy with 0.8231 QWK respectively, outperforming representative CNN, transformer, and foundation-model baselines while remaining within the compute envelope of standard single-stream backbones. Counter-intuitively, performance is preserved or improved on the lowest-quality stratum (77.61% on EyeQ-Reject; 82.21% on DDR-Reject, exceeding the Good-quality accuracy on the same dataset), and a Shuffled-IQA counterfactual confirms that QGDR exploits the semantic content of quality information rather than a generic auxiliary signal. Test-only evaluation on the external IDRiD and DeepDRiD cohorts confirms cross-dataset generalization. By treating image quality as guidance rather than as a filter, QGDR preserves screening coverage without sacrificing diagnostic reliability.

## 1 Introduction

Diabetic retinopathy (DR) is a leading cause of preventable vision loss worldwide, affecting approximately 93 million people (1). Early detection and accurate grading are essential for timely intervention (2, 3), and deep learning has driven substantial progress in automated DR diagnosis (2, 4–6). Existing methods fall into two broad categories. Image-level approaches train on severity labels via classification (7, 8) or hierarchical multi-task frameworks (4, 9, 10), whereas lesion-level approaches localize pathologies through attention mechanisms (11, 12) or contrastive learning (13). Recent advances include vision transformers (14), foundation models (15), and strategies for class imbalance such as long-tailed learning (16) and hierarchical knowledge guidance (17). Despite strong performance on curated datasets, clinical deployment remains challenging because of accuracy degradation on the low-quality fundus images that dominate real-world screening programs (18, 19).

**Figure 1:**
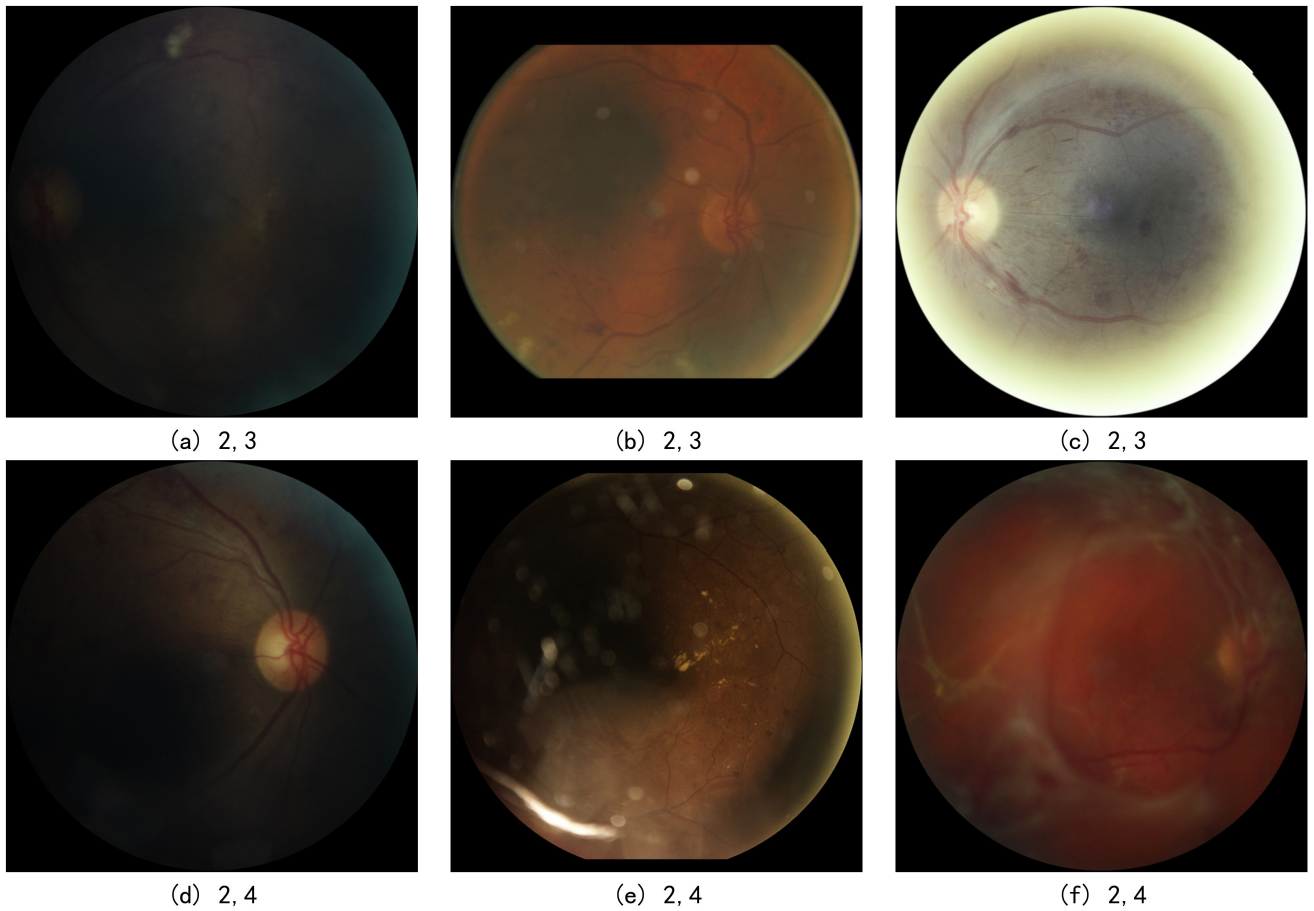
Fundus images rated as Reject quality that nonetheless contain diagnostically valuable DR features. **(a–c)** DR grade 3; **(d–f)** DR grade 4. Pathological features remain clinically informative despite low image quality, illustrating the quality-severity coupling phenomenon.

A fundamental challenge arises from the intrinsic relationship between image quality and disease severity. As illustrated in Figure 1, advanced DR stages frequently coincide with poor image quality, because pathological changes such as vitreous hemorrhage and proliferative tissue themselves degrade the imaging optics (20, 21). We term this phenomenon *quality-severity coupling*. Critically, such degraded images still carry diagnostic information: microaneurysms, exudates, and neovascularization often remain visible despite quality loss. This coupling challenges the conventional paradigm of treating IQA as a preprocessing step, because filtering or enhancing low-quality images risks discarding or distorting the most clinically significant cases. In parallel, fundus IQA itself has evolved from structure-based approaches using vessel segmentation (22) to learning-based methods built on deep networks. Fu et al. (23) established the EyeQ benchmark with standardized quality levels, and subsequent works incorporated multi-scale features (19, 24), transformer architectures (21, 25), and contrastive learning (26); recent surveys provide a broader account of IQA methods and clinical applications (18, 27). However, existing IQA methods typically operate as independent preprocessing or filtering modules and do not exploit the intrinsic correlation between quality assessment and disease grading.

**Figure 2:**
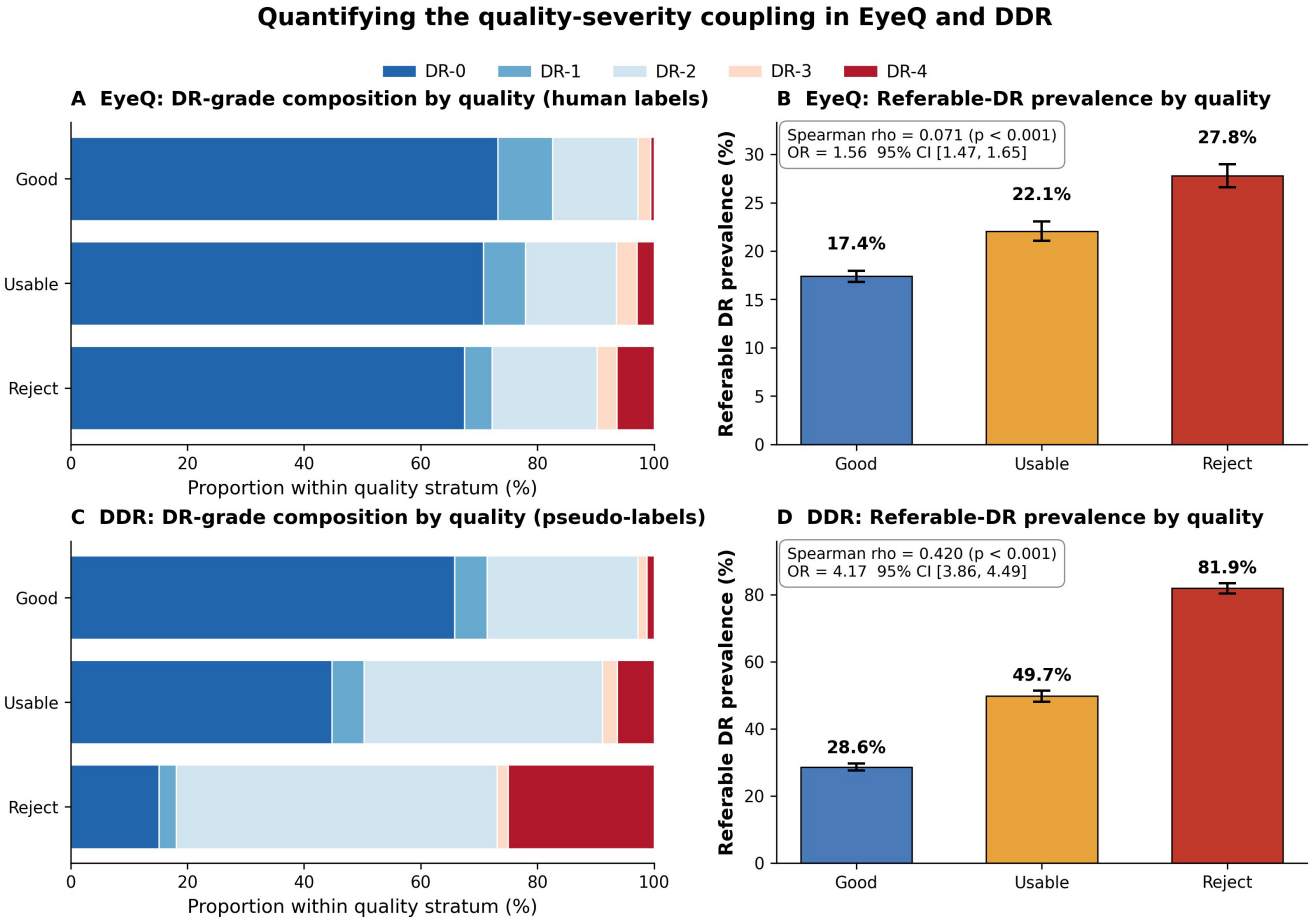
Dataset-level quantification of the quality-severity coupling phenomenon. **(A, C)** DR-grade composition within each IQA stratum (Good/Usable/Reject) for EyeQ (top, human-labeled IQA) and DDR (bottom, IQA labels generated by the Stage-1 model; reliability validated *post-hoc* in Section 3.1); error bars denote 95% binomial CIs for the leading DR-0 proportion. **(B, D)** Referable-DR (grades ≥2) prevalence per quality stratum with 95% binomial CIs. Insets report the Spearman rank correlation ρ between quality and DR severity, and the odds ratio (OR) of referable DR in Reject vs. Good strata with 95% CIs. Both datasets show a statistically significant association (*p* < 0.001), with the effect substantially stronger on DDR (ρ = 0.420, OR = 4.17) than on EyeQ (ρ = 0.071, OR = 1.56).

To quantify this phenomenon at the dataset level, we analyze the joint distribution of image quality and DR severity in EyeQ and DDR (Figure 2). On EyeQ, the prevalence of referable DR (grades ≥2) rises from 17.4% in Good-quality images to 27.8% in Reject-quality images (Spearman ρ = 0.071, *p* < 0.001; odds ratio [OR] 1.56, 95% CI [1.47, 1.65]). The coupling is markedly stronger on DDR, where referable-DR prevalence rises from 28.6% (Good) to 81.9% (Reject) (ρ = 0.420, *p* < 0.001; OR = 4.17, 95% CI [3.86, 4.49]). Both datasets therefore show a statistically significant association between lower image quality and higher DR severity, with DDR exhibiting substantially stronger coupling, consistent with its more heterogeneous real-world acquisition conditions. This quantitative evidence motivates the central premise of the present work: low-quality images cannot be safely treated as preprocessing failures, because they are disproportionately enriched for clinically critical severe DR.The question, then, is not whether to use these images but how to process them. Existing paradigms answer this question implicitly, and we examine their limitations next.

Current approaches share inherent limitations in addressing this coupling. Enhancement-based methods, including classical histogram equalization (28), GAN-based restoration (29, 30), and domain adaptation (31, 32), risk distorting subtle pathological features such as microaneurysms and may introduce artifacts; low-illumination enhancement (33) encounters analogous difficulties in preserving fine diagnostic detail. Quality-filtering pipelines (18, 34) systematically discard severe DR cases whose quality degradation is itself caused by the disease. Multi-task methods (35, 36) jointly optimize IQA and DR classification, but treat them as parallel objectives sharing a common representation, without modeling how quality states should condition disease-specific feature learning. The underlying limitation is shared: enhancement modifies inputs before diagnosis, filtering bypasses diagnosis altogether, and joint multi-task learning leaves the IQA branch passive. None of these paradigms uses quality as an active guidance signal that propagates through the diagnostic pipeline.

From this analysis we identify three gaps motivating the present work. First, no existing mechanism integrates quality features into spatial attention to support quality-aware representation learning. Second, existing methods lack adaptive grading strategies that adjust their behavior according to image quality. Third, the multi-scale nature of DR lesions, whose sizes range from tiny microaneurysms to extensive hemorrhages, remains insufficiently exploited. Several architectural primitives are well suited to bridging these gaps. Feature pyramid networks (37) and the bidirectional feature pyramid network (BiFPN) (38) enable efficient multi-scale fusion through learnable weighted connections. Mixture-of-experts architectures (39, 40) dynamically route inputs to specialized sub-networks via learned gating functions, and multi-task learning (41) exploits complementary signals from related tasks. However, prior joint IQA-DR formulations (35, 36) do not use quality predictions to modulate expert routing or attention for quality-adaptive multi-scale processing.

To address these gaps, we propose QGDR, a quality-guided dynamic routing framework that integrates IQA into DR grading through end-to-end collaborative learning. The core idea is to transform quality assessment from a binary filtering decision into a continuous guidance signal that simultaneously shapes feature learning and expert routing. Specifically, a multi-level IQA module extracts hierarchical quality features across backbone stages, which then guide spatial attention through learnable context gating. An adaptive gated fusion mechanism selects scale-specialized experts conditioned on the predicted quality state (40): high-quality images preferentially activate fine-scale experts for subtle lesion detection, while degraded images rely more on coarse-scale experts for robust global pattern recognition. Temperature scaling (42) and probability-space ensemble fusion (43) stabilize this routing.

The main contributions of this work are summarized as follows:

- We identify and quantitatively characterize the quality-severity coupling phenomenon, and re-frame IQA from a preprocessing filter to a continuous guidance signal that enables effective use of clinically informative low-quality images.
- We design a quality-modulated attention mechanism that injects hierarchical quality features into spatial attention through learnable context gating, supporting quality-aware feature learning.
- We introduce an adaptive gated fusion mechanism that routes inputs to scale-specialized experts conditioned on the predicted quality state, yielding robust DR grading across diverse quality strata.

Extensive experiments on EyeQ and DDR, together with test-only evaluation on the external IDRiD and DeepDRiD cohorts, show that QGDR attains competitive performance compared with state-of-the-art methods and is notably robust across quality strata.

## 2 Methods

### 2.1 Framework overview

**Figure 3:**
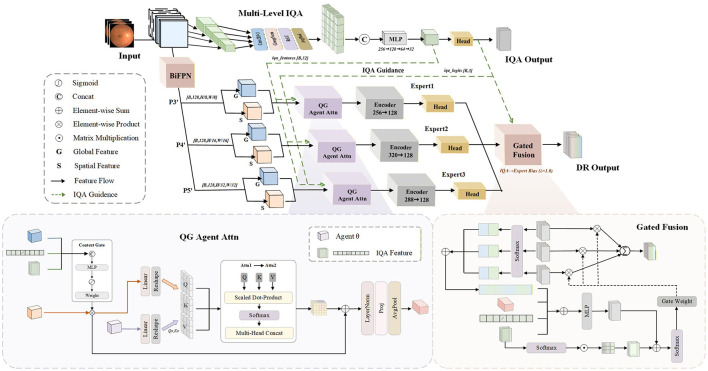
Overview of the QGDR framework. (1) Swin Transformer backbone extracts hierarchical features across stages S1–S4 (channels [96, 192, 384, 768]); (2) multi-level IQA module produces quality predictions and 32-dimensional embeddings **f**_IQA_ (**top**); (3) BiFPN aggregates features from S2–S4 into three scales P3'–P5' (**left inset**: context gate); (4) three scale-specialized experts apply quality-modulated agent attention (**middle inset**: agent tokens with quality-guided context gating); (5) adaptive gating network weights expert contributions by quality state (**right inset**: softmax-normalized fusion). Green dashed arrows denote IQA guidance flow.

As illustrated in Figure 3, the proposed QGDR framework integrates IQA with DR grading through collaborative multi-scale learning. Given a fundus image dataset $X={\left\{x_{i}\right\}}_{i=1}^{N}$ with quality labels $Y^{\text{IQA}}={\left\{y_{i}^{\text{IQA}}\right\}}_{i=1}^{N}$ ($y_{i}^{\text{IQA}}\in \left\{0,1,2\right\}$ for good, usable, reject) and DR grading labels $Y^{\text{DR}}={\left\{y_{i}^{\text{DR}}\right\}}_{i=1}^{N}$ ($y_{i}^{\text{DR}}\in \left\{0,1,2,3,4\right\}$ for no DR, mild, moderate, severe, and proliferative DR), the framework adopts a two-stage training strategy (44). Stage 1 pretrains a Swin Transformer backbone (45) for IQA to learn quality-aware representations. Stage 2 extends the pretrained backbone for DR grading with quality-guided multi-scale collaborative learning.

The complete architecture operates as follows. The Swin Transformer backbone extracts hierarchical features across four stages (S1–S4) with channel dimensions [96, 192, 384, 768]. The multi-level IQA module processes these features and outputs quality predictions ŷ^IQA^ along with 32-dimensional quality embeddings ${\text{f}}_{\text{IQA}}\in {\mathbb{R}}^{32}$. A BiFPN module (38) aggregates features from S2, S3, and S4 into three spatial resolutions P3', P4', and P5' with dimensions [*B*, 128, *H*/8, *W*/8], [*B*, 128, *H*/16, *W*/16], and [*B*, 128, *H*/32, *W*/32], respectively. Three scale-specialized experts process these multi-scale features, where each expert employs a quality-modulated attention mechanism that integrates spatial features, global context, and IQA guidance through learnable agent tokens (46) and context gating. An adaptive gating network dynamically weights the expert predictions based on quality states (40), producing the final DR prediction ŷ^DR^. The framework is jointly optimized via a multi-task loss (41) combining IQA classification, individual expert losses, ensemble prediction, gate balance regularization, and multi-scale diversity supervision.

### 2.2 Multi-scale feature fusion and quality-modulated attention

**Figure 4:**
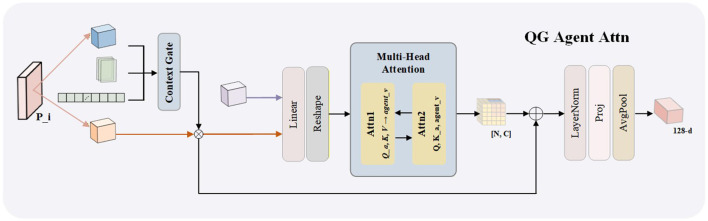
Multi-scale feature fusion and quality-modulated agent attention. BiFPN aggregates features from S2–S4 into three resolutions P3'–P5'. Quality-conditioned context gating modulates spatial features, after which learnable agent tokens enable efficient bidirectional feature aggregation.

To capture DR-related lesions that exhibit multi-scale characteristics while maintaining quality awareness, we employ a two-stage processing pipeline: bidirectional feature pyramid network for multi-scale feature aggregation, followed by quality-modulated agent attention for efficient feature learning, as illustrated in Figure 4.

#### 2.2.1 Bidirectional feature pyramid network

We employ BiFPN (38) to aggregate multi-scale features from backbone stages S2, S3, and S4, denoted as $\left\{P_{3}^{\text{in}},P_{4}^{\text{in}},P_{5}^{\text{in}}\right\}$ with spatial resolutions *H*/8 × *W*/8, *H*/16 × *W*/16, and *H*/32 × *W*/32, respectively. BiFPN performs bidirectional top-down and bottom-up feature fusion with learnable weights, enabling effective information exchange across scales. After *L* = 2 layers of BiFPN processing, we obtain enhanced multi-scale features $\left\{P_{3}^{\text{out}},P_{4}^{\text{out}},P_{5}^{\text{out}}\right\}$ with unified channel dimension *C* = 128. Each output feature is decomposed into spatial features ${\text{F}}_{\text{spatial}}^{\left(i\right)}\in {\mathbb{R}}^{B\times N_{i}\times C}$ and global features ${\text{f}}_{\text{global}}^{\left(i\right)}\in {\mathbb{R}}^{B\times C}$ via global average pooling, where *N*_*i*_ denotes the number of spatial positions at resolution *i*.

#### 2.2.2 Quality-conditioned context gating

To incorporate quality awareness into spatial feature processing, we aggregate quality-related information from multiple sources. For each scale *i*, global features ${\text{f}}_{\text{global}}^{\left(i\right)}$, IQA embeddings **f**_IQA_, and quality logits ŷ^IQA^ are concatenated to form a quality context vector:


$$\begin{array}{l} {\text{c}}_{\text{quality}}^{\left(i\right)}=\left[{\text{f}}_{\text{global}}^{\left(i\right)},{\text{f}}_{\text{IQA}},{ŷ}^{\text{IQA}}\right] \end{array}$$ (1)

A context gating network transforms this quality context into spatial modulation weights:


$$\begin{array}{l} {\text{g}}^{\left(i\right)}=\sigma \left(\text{MLP}\left({\text{c}}_{\text{quality}}^{\left(i\right)}\right)\right) \end{array}$$

$$\begin{array}{l} {\text{F}}_{\text{mod}}^{\left(i\right)}={\text{F}}_{\text{spatial}}^{\left(i\right)}⊙{\text{g}}^{\left(i\right)} \end{array}$$ (2)

where σ(·) denotes sigmoid activation and ⊙ represents element-wise multiplication. This modulation adaptively emphasizes or suppresses spatial features based on image quality states, enabling quality-aware feature learning.

#### 2.2.3 Quality-modulated agent attention

To efficiently aggregate spatial features while maintaining quality awareness, we adopt an agent attention mechanism inspired by Han et al. (46). We introduce learnable agent tokens **A**^(*i*)^ ∈ ℝ^*M*×*C*^ (where *M* = 49 ≪ *N*_*i*_) as intermediaries that reduce computational complexity from $O\left(N_{i}^{2}\right)$ to *O*(*N*_*i*_*M*). Unlike standard agent attention, our mechanism incorporates quality-modulated spatial features ${\text{F}}_{\text{mod}}^{\left(i\right)}$ as input, enabling quality-aware feature aggregation. The agent tokens first aggregate information from the modulated spatial features through attention, then redistribute the aggregated information back to spatial positions. The final attended features for scale *i* are obtained through global average pooling:


$$\begin{array}{l} {\text{f}}_{i}^{\text{attended}}=\text{GAP}\left({\text{F}}_{\text{attended}}^{\left(i\right)}\right) \end{array}$$ (3)

This design achieves efficient quality-aware multi-scale feature learning by combining BiFPN-based feature aggregation with quality-conditioned modulation and agent-based attention.

### 2.3 Multi-scale expert ensemble with adaptive gating

After quality-modulated attention processing, we design three scale-specialized experts that independently process the attended features from different spatial resolutions, followed by an IQA-guided adaptive gating mechanism for quality-adaptive ensemble predictions.

#### 2.3.1 Scale-specialized experts

Three experts independently process attended features at different spatial scales, each optimized for detecting lesions of specific sizes:


$$\begin{array}{l} {\text{z}}_{i}={\text{Classifier}}_{i}\left({\text{Encoder}}_{i}\left({\text{f}}_{i}^{\text{attended}}\right)\right),\;i\in \left\{3,4,5\right\} \end{array}$$ (4)

Expert 1 (P3', *H*/8 × *W*/8) focuses on fine-grained details for detecting tiny microaneurysms. Expert 2 (P4', *H*/16 × *W*/16) captures medium-scale features for hemorrhages and exudates. Expert 3 (P5', *H*/32 × *W*/32) processes coarse-scale features for global pathological patterns. Each expert employs a dedicated encoder with hidden dimensions [256, 320, 288], respectively.

#### 2.3.2 IQA-guided adaptive gating

A key component is the quality-guided gating mechanism that dynamically weights expert contributions based on image quality. The gating network takes as input the concatenation of attended features, quality information, and expert confidence measures:


$$\begin{array}{l} {\text{c}}_{\text{gate}}=\left[{\text{f}}^{\text{avg}},{\text{f}}_{\text{IQA}},{\text{h}}_{\text{conf}},{ŷ}^{\text{IQA}}\right] \end{array}$$ (5)

where ${\text{f}}^{\text{avg}}=\frac{1}{3}\overset{5}{\underset{i=3}{\sum }}{\text{f}}_{i}^{\text{attended}}$ represents averaged attended features, and ${\text{h}}_{\text{conf}}\in {\mathbb{R}}^{6}$ aggregates expert confidence measures derived from prediction entropy and maximum probability. To enable quality-guided expert selection, we introduce a learnable quality-to-expert bias that adjusts gating logits based on quality states:


$$\begin{array}{l} \text{g}=\text{MLP}\left({\text{c}}_{\text{gate}}\right)\\ {\text{b}}_{\text{IQA}}={\text{p}}^{\text{IQA}}{\text{W}}_{\text{IQA}}\\ \;\text{w}=\text{softmax}\left(\frac{\text{g}+\lambda \cdot {\text{b}}_{\text{IQA}}}{\tau }\right) \end{array}$$ (6)

where **p**^IQA^ = softmax(ŷ^IQA^) is the quality probability distribution, ${\text{W}}_{\text{IQA}}\in {\mathbb{R}}^{3\times 3}$ is a learnable quality-to-expert bias matrix, λ = 0.7 controls the quality guidance strength, and τ = 1.0 is the temperature parameter (42). The final ensemble prediction is obtained through weighted probability-space fusion (43):


$$\begin{array}{l} {\text{p}}_{\text{final}}=\overset{5}{\underset{i=3}{\sum }}w_{i-2}\cdot \text{softmax}\left({\text{z}}_{i}\right) \end{array}$$ (7)

This design encourages the model to assign higher weights to fine-scale experts for high-quality images with clear details, while favoring coarse-scale experts for degraded images where global patterns are more reliable.

#### 2.3.3 Multi-task loss function

The framework is optimized through a multi-task learning strategy (41, 47) with a composite loss function:


$$\begin{array}{l} L_{\text{total}}=\lambda_{\text{IQA}}L_{\text{IQA}}+\lambda_{\text{experts}}\frac{1}{3}\overset{3}{\underset{i=1}{\sum }}L_{{\text{expert}}_{i}}\\ \;+\lambda_{\text{final}}L_{\text{final}}+\lambda_{\text{balance}}L_{\text{balance}}\\ \;+\lambda_{\text{diversity}}L_{\text{diversity}} \end{array}$$ (8)

where $L_{\text{IQA}}$ supervises quality classification, $L_{{\text{expert}}_{i}}$ supervises individual expert DR predictions, $L_{\text{final}}$ supervises the ensemble prediction, $L_{\text{balance}}$ encourages balanced expert utilization, and $L_{\text{diversity}}$ promotes multi-scale specialization. The loss weights are set as λ_IQA_ = 0.3, λ_experts_ = 0.5, λ_final_ = 0.8, λ_balance_ = 0.05, and λ_diversity_ = 0.22.

#### 2.3.4 Focal loss for class imbalance

To address class imbalance in DR datasets, we employ Focal Loss (48) for all classification tasks:


$$\begin{array}{l} L_{\text{FL}}=-\alpha_{t}{\left(1-p_{t}\right)}^{\gamma }\log\left(p_{t}\right) \end{array}$$ (9)

where *p*_*t*_ is the predicted probability for the ground truth class, α_*t*_ is the class-specific weight computed from training data distribution, and γ = 1.5 is the focusing parameter that down-weights easy examples.

#### 2.3.5 Gate balance loss

To prevent degenerate solutions where the gating network assigns all weight to a single expert (40), we introduce a balance regularization:


$$\begin{array}{l} L_{\text{balance}}=\overset{3}{\underset{i=1}{\sum }}{\left({\bar{w}}_{i}-\frac{1}{3}\right)}^{2} \end{array}$$ (10)

where ${\bar{w}}_{i}=\frac{1}{B}\overset{B}{\underset{b=1}{\sum }}w_{i}^{\left(b\right)}$ is the mean gate weight for expert *i* across the batch.

#### 2.3.6 Multi-scale diversity loss

To encourage expert specialization based on DR severity, we introduce a diversity loss with soft target weights:


$$\begin{array}{l} L_{\text{diversity}}=\frac{1}{B}\overset{B}{\underset{b=1}{\sum }}∥{\text{w}}^{\left(b\right)}-{\text{w}}_{\text{target}}^{\left(b\right)}{∥}_{2}^{2} \end{array}$$ (11)

where **w**^(*b*)^ ∈ ℝ^3^ are the predicted gate weights. The target weights ${\text{w}}_{\text{target}}^{\left(b\right)}$ are determined by DR severity to embed clinical domain knowledge: early-stage DR (grades 0–1) predominantly targets fine-scale Expert 1 with [0.60, 0.25, 0.15], moderate DR (grades 2–3) targets medium-scale Expert 2 with [0.15, 0.65, 0.20], and advanced DR (grade 4) targets coarse-scale Expert 3 with [0.10, 0.20, 0.70]. This soft supervision guides meaningful multi-scale specialization while allowing flexibility through the relatively small weight λ_diversity_ = 0.20.

### 2.4 Datasets and evaluation metrics

We conduct experiments on three datasets for model training and evaluation. For performance assessment, we employ Overall Accuracy (OA) and Quadratic Weighted Kappa (QWK) as primary metrics for DR five-class grading.

**Figure 5:**
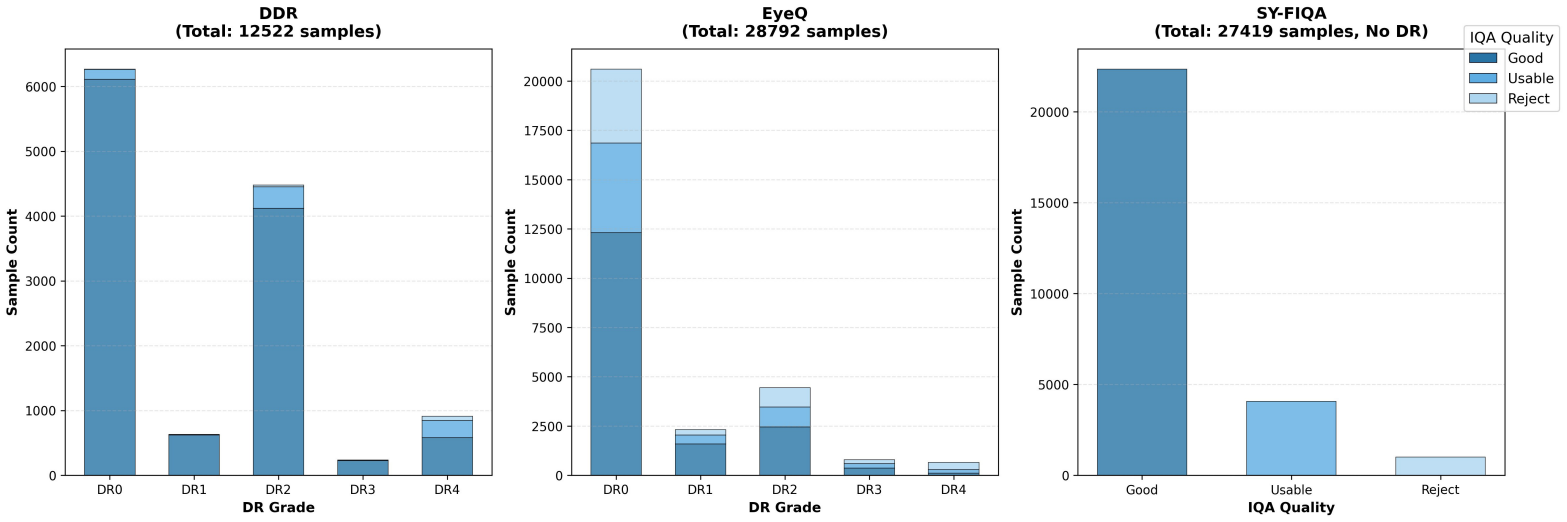
IQA-DR label distribution across three datasets. DDR and EyeQ provide paired IQA and DR annotations for joint training. Bars are stacked to show IQA quality distribution (Good/Usable/Reject) within each DR grade. SY-FIQA contains only IQA labels for Stage-1 fine-tuning.

**EyeQ Dataset** (23): EyeQ is a large-scale benchmark derived from the EyePACS screening program, comprising 28,792 retinal images with paired IQA labels (Good/Usable/Reject) and DR grading labels (grades 0–4). As shown in Figure 5, the dataset exhibits significant class imbalance, with normal cases (DR0) accounting for approximately 70% of samples, while severe cases (DR3–4) represent less than 5%. The quality-severity coupling phenomenon is evident in EyeQ, where advanced DR grades show higher proportions of low-quality images.

**DDR Dataset** (49): The Diabetic Retinopathy Dataset (DDR) contains 13,673 fundus images from 147 hospitals in China, including six categories (DR grades 0–4 and ungradable). In our experiments, we use the gradable subset comprising 12,522 images with DR grades 0–4 for training and evaluation.

**SY-FIQA Dataset**: To enhance the IQA module's generalization, we incorporate SY-FIQA, a real-world dataset collected from Zhongshan Ophthalmic Center at Sun Yat-sen University. The dataset comprises 27,419 fundus images with quality annotations (Good/Usable/Reject) labeled by three clinical experts. The distribution includes 22,346 Good-quality images (81.5%), 4,074 Usable images (14.9%), and 999 Reject images (3.6%). This dataset is used for fine-tuning the Stage-1 IQA model to improve generalization across diverse clinical conditions.

EyeQ and DDR are used for joint IQA-DR training in Stage 2, while SY-FIQA provides real-world quality diversity for fine-tuning the IQA module in Stage 1. Figure 5 shows the label distributions across the three datasets. The reliability of the pseudo-IQA labels generated for DDR is validated *post-hoc* in Section 3.1.

### 2.5 Implementation details

#### 2.5.1 Two-stage training strategy

The two-stage design is motivated by the asymmetric optimization difficulty of IQA and DR grading and by the need for stable quality-aware representations before downstream coupling. Joint optimization from scratch requires the backbone to simultaneously acquire (a) quality-discriminative features sensitive to image-level noise, illumination, and clarity, and (b) lesion-discriminative features sensitive to small focal abnormalities, with no prior on the relative magnitude of the two losses. Under this regime, one task may dominate and yield a backbone biased toward a single objective. Pretraining the backbone on IQA in Stage 1 establishes quality-aware representations first; lower layers are subsequently frozen and upper layers refined in Stage 2, where the DR grading components are introduced. This curriculum is consistent with prior evidence that pretraining on auxiliary tasks before introducing the primary task improves convergence and final performance in multi-task settings (44, 47). The ablation study (Section 3.4) supports this choice empirically: removing IQA pretraining (w/o IQA Pretrain in Table 1) reduces accuracy by 8.40 points on EyeQ and 5.16 points on DDR.

**Table 1:** Ablation of QGDR on EyeQ and DDR, including the Shuffled-IQA counterfactual on DDR.

Configuration	EyeQ			DDR		
	Acc	QWK	AUC	Acc	QWK	AUC
w/o IQA Pretrain	0.6992	0.6134	0.8242	0.7569	0.7815	0.8858
+ IQA Pretrain, w/o Multi-Level IQA	0.7279	0.6110	0.8195	0.7191	0.7432	0.8407
+ Multi-Level IQA, w/o Quality Mod.	0.5011	0.5437	0.8147	0.6877	0.7372	0.8566
+ Quality Mod., w/o BiFPN	0.6956	0.5611	0.7926	0.7130	0.7447	0.8524
+ BiFPN, w/o Agent Attention	0.6940	0.6216	0.8139	0.7361	0.7551	0.8588
+ Agent Attention, w/o Multi-Scale Experts	0.7354	0.6337	0.8310	0.7257	0.7359	0.8443
+ Multi-Scale Experts, w/o IQA-Guided Gating	0.7404	0.6086	0.8180	0.7108	0.7494	0.8301
Full Model (QGDR)	**0.7832**	**0.6863**	**0.8501**	**0.8085**	**0.8231**	**0.9015**
Shuffled-IQA (counterfactual)^†^	–	–	–	0.7598	0.7492	0.8617

AUC denotes the macro-averaged 5-class AUC. Each row progressively adds the named module. Best results in bold.

^†^ Shuffled-IQA: pseudo-IQA labels of the DDR training set are randomly permuted while preserving the marginal class distribution; all other hyperparameters match the Full Model. The drop from 0.8085 (Full) to 0.7598 (Shuffled) confirms that the IQA pathway exploits the content of the pseudo-labels rather than acting as a generic auxiliary supervision channel.

In Stage 1, we pretrain the IQA module using the Swin Transformer backbone on EyeQ for 80 epochs with learning rate 1 × 10^−4^ and AdamW optimizer. The pretrained model is then fine-tuned on SY-FIQA to improve generalization to diverse clinical scenarios. This fine-tuned model provides quality-aware feature representations for Stage 2 and generates pseudo IQA labels for DDR, which lacks ground-truth quality annotations. The reliability of these pseudo-labels is validated *post-hoc* in Section 3.1.

In Stage 2, the DR grading network is initialized with the pretrained backbone, while new components (BiFPN, attention modules, experts, and gating network) are randomly initialized. The first two backbone layers are frozen to preserve quality-aware representations. The network is trained for 100 epochs with learning rate 5 × 10^−5^, using layer-wise learning rate decay (factor 0.95) for the backbone. A 5-epoch warmup phase is employed.

#### 2.5.2 Data preprocessing and augmentation

**Figure 6:**
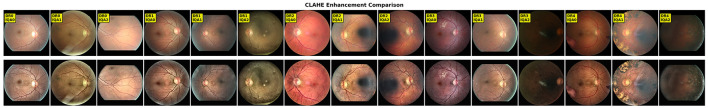
Comparison between original and CLAHE-enhanced fundus images. **Top** row shows original images, **bottom** row shows corresponding enhanced versions across different quality grades and DR severity levels.

All images are resized to 224 × 224 pixels. We apply CLAHE (28) to all images uniformly as a deterministic, parameter-free preprocessing step rather than as an adaptive quality-restoration mechanism. Two design considerations motivate this choice. First, our framework deliberately preserves quality variation as a learning signal. Heavy enhancement methods, such as GAN-based restoration (29, 30), would suppress the quality cues that the IQA module relies on, thereby undermining the central paradigm. CLAHE, by contrast, performs only local contrast normalization within tile-based histograms and does not synthesize new content, leaving quality differences detectable. Second, CLAHE improves the visibility of low-contrast vessels and small lesions such as microaneurysms and hard exudates, which are clinically informative regardless of quality stratum. This provides a uniform improvement to all images without distorting their relative quality ordering. Following preprocessing, dataset-specific normalization parameters are computed from CLAHE-enhanced samples. Data augmentation includes random horizontal flipping and rotation (±10°), with additional color augmentation for minority classes (DR3–4). Mixup (α = 0.2) is applied after warmup. A visual comparison of CLAHE outputs across quality strata is shown in Figure 6.

#### 2.5.3 Soft balanced sampling

To address class imbalance while preserving natural distribution characteristics, we employ a soft balanced sampling strategy:


$$\begin{array}{l} w_{c}=\left(1-\beta \right)\cdot w_{c}^{\text{real}}+\beta \cdot w_{c}^{\text{uniform}} \end{array}$$ (12)

where $w_{c}^{\text{real}}$ is the natural distribution weight, $w_{c}^{\text{uniform}}=1/C$ is the uniform weight, and β = 0.3 controls balance strength.

#### 2.5.4 Network architecture

We employ Swin Transformer Tiny (45) as the backbone with channel dimensions [96, 192, 384, 768]. The BiFPN module uses 128 channels with 2 layers. Three experts have hidden dimensions [256, 320, 288] with dropout rate 0.2. The agent attention employs 4 heads with 49 agent tokens. The gating network uses hidden dimension 128 with temperature τ = 1.0 and quality guidance strength λ = 0.7.

#### 2.5.5 Loss configuration

Focal Loss (48) is employed with γ = 1.5. Multi-task loss weights are λ_IQA_ = 0.3, λ_experts_ = 0.5, λ_final_ = 0.8, λ_balance_ = 0.05, and λ_diversity_ = 0.20.

All models are trained with batch size 32 on a single NVIDIA GeForce RTX 2080 Ti GPU using AdamW optimizer. Dataset splitting employs stratified random sampling with 20% allocated to validation.

## 3 Results

### 3.1 Stage-1 IQA and pseudo-label validation

DDR does not provide ground-truth IQA annotations, and the Stage-2 pipeline of QGDR therefore relies on pseudo-IQA labels generated by a single Stage-1 model (Swin-T) on each DDR image. The validity of the downstream quality-severity coupling analysis depends directly on the reliability of these pseudo-labels. We address this dependency through three lines of evidence, presented in turn below. Throughout this subsection, the additional backbones ConvNeXt-T (50) and DINOv2 ViT-S/14 (51) are used solely as architecturally distinct cross-checks; they are not part of QGDR.

**(i) In-domain competence of the Stage-1 model** A necessary first condition is that the Stage-1 model is itself a competent IQA classifier on its training distribution. Table 2 reports EyeQ test-set IQA performance for three candidate backbones: Swin-T (45) (hierarchical Transformer, ImageNet-1K supervised), ConvNeXt-T (modernized CNN, ImageNet-1K supervised), and DINOv2 ViT-S/14 (plain ViT, self-supervised on LVD-142M). All three reach Acc ≥0.9123 and QWK ≥0.9268, with ConvNeXt-T marginally ahead. The narrow gap across architectural families indicates that the IQA task is not strongly architecture-dependent under sufficient supervision, and that the Swin-T backbone used in QGDR is a strong supervised baseline rather than a one-of-a-kind choice.

**Table 2:** Stage-1 IQA performance on the EyeQ in-domain test set.

Model	Pretraining	Params (M)	Acc	QWK	AUC
Swin-T (used in QGDR) (45)	ImageNet-1K (sup.)	27.7	0.9310	0.9401	0.9850
ConvNeXt-T (50)	ImageNet-1K (sup.)	28.0	**0.9382**	**0.9479**	**0.9868**
DINOv2 ViT-S/14 (51)	DINOv2 SSL (LVD-142M)	21.7	0.9123	0.9268	0.9846

Best per-column values in bold.

**(ii) External transfer of Stage-1 IQA features** Strong in-domain accuracy alone does not preclude overfitting to EyeQ-specific imaging characteristics. To examine whether the Stage-1 representation captures genuine quality semantics, we evaluate the same three backbones, each completing the full Stage-1 procedure (EyeQ training followed by SY-FIQA fine-tuning), on FQS (*N* = 2,246) under a test-only protocol. Despite the substantial domain shift, all three backbones retain reasonable agreement with expert IQA annotations (QWK ≥0.6630, Table 3). The goal here is not to chase state-of-the-art on FQS but to confirm that the Stage-1 features carry transferable quality information rather than dataset-specific artifacts.

**Table 3:** Stage-1 IQA evaluation on the FQS expert-annotated set (*N* = 2,246) under a test-only protocol; each model is trained on EyeQ only.

Model	Acc (95% CI)	QWK (95% CI)
Swin-T (used in QGDR) (45)	0.6310 [0.6120, 0.6510]	0.6630 [0.6410, 0.6850]
ConvNeXt-T (50)	0.6570 [0.6380, 0.6770]	0.6940 [0.6730, 0.7140]
DINOv2 ViT-S/14 (51)	**0.6830 [0.6650, 0.7030]**	**0.7100 [0.6850, 0.7330]**

95% confidence intervals are obtained from 1,000 bootstrap resamples. Best per-column values in bold.

**(iii) Cross-estimator consistency of the pseudo-labels on DDR** Because DDR has no IQA ground truth, the most direct test of pseudo-label reliability is whether multiple, architecturally distinct estimators converge on the same labels. We treat Swin-T, ConvNeXt-T, and DINOv2 as three independent estimators and quantify their agreement on DDR (the target distribution for the pseudo-labels) and on FQS (where expert labels are available, used here as a sanity reference). Table 4 reports pairwise statistics. All three pairs reach *P*_*o*_≥0.79 and QWK ≥0.79 on both datasets, with low Jensen-Shannon divergence and high Pearson correlation of the soft predictions. Aggregated across all three estimators, multi-rater statistics on DDR yield Fleiss' κ = 0.7843 and ordinal Krippendorff's α = 0.9355. Three independently parameterized backbones therefore converge on essentially the same quality assignments for DDR images, supporting the view that the pseudo-labels reflect image-level quality signal rather than single-model idiosyncrasies.

**Table 4:** Pairwise agreement metrics among the three models on DDR (pseudo-label target) and FQS (expert-labeled set, *N* = 2,246).

Model pair	DDR				FQS			
	*P*_*o*_	QWK	JS div.	Pearson *r*	*P*_*o*_	QWK	JS div.	Pearson *r*
Swin-T vs. ConvNeXt-T	0.8730	0.8830	0.0926	**0.8760**	**0.8540**	**0.8540**	**0.0872**	**0.8620**
Swin-T vs. DINOv2	0.8620	0.8760	**0.0826**	0.8680	0.7950	0.8070	0.1097	0.8180
ConvNeXt-T vs. DINOv2	**0.8780**	**0.8900**	0.1363	0.8460	0.8160	0.8290	0.1495	0.7930

Each model follows the complete Stage-1 procedure (EyeQ training followed by SY-FIQA fine-tuning). On FQS, the models are applied under a test-only protocol to compute agreement with expert labels; on DDR, pseudo-labels are generated and pairwise agreement among the three models is computed. 95% confidence intervals are obtained from 1,000 bootstrap resamples. Best per-column values in bold.

A residual concern is that high overall agreement could mask a failure mode specific to severe-DR strata, which are precisely the cases where the quality-severity coupling is most pronounced and where pseudo-label noise would be most consequential for the downstream analysis. Table 5 addresses this by stratifying the multi-rater agreement on DDR by DR grade. Across the full set, all three estimators produce identical labels on 80.7% of samples, at least two of three agree on a further 19.1%, and only 0.2% of cases see all three estimators disagree. Within each DR stratum, Fleiss' κ remains ≥0.7114, ordinal Krippendorff's α exceeds 0.89, and the proportion of samples receiving the same label from a majority (at least two of three) of the backbones is ≥99.5% throughout, including DR3 (severe NPDR) and DR4 (PDR). Pseudo-label noise is therefore not concentrated in the clinically most demanding strata, and the validity of the coupling analysis is preserved exactly where it matters most.

**Table 5:** DR-stratified pseudo-label consistency on DDR across the three Stage-1 backbones (Swin-T, ConvNeXt-T, DINOv2 ViT-S/14).

DR Stratum	*N*	Fleiss' κ	Krippendorff's α (ord.)	All-3 agree (%)	Majority (≥2) (%)
All DDR	12,522	0.7843	0.9355	80.7	99.8
DR=0 (No DR)	6,266	0.7538	0.9006	83.5	99.9
DR=1 (Mild)	630	0.7658	0.9273	81.0	99.5
DR=2 (Moderate)	4,477	0.7654	0.9341	77.0	99.7
DR=3 (Severe)	236	0.7114	0.8965	73.3	100.0
DR=4 (PDR)	913	0.7442	0.9137	80.7	99.8

“All-3 agree” is the percentage of samples on which all three backbones output the same label; “Majority (≥2)” is the percentage on which at least two of three agree.

### 3.2 Comparison with state-of-the-art methods

We benchmark QGDR against representative DR grading approaches on EyeQ and DDR. The compared methods include classic CNN backbones [ResNet-50 (52), DenseNet-121 (53), EfficientNet-B4 (54)], advanced vision architectures [CoAtNet (55), ConvNeXt (50), PVTv2 (56), ViT-S (57)], multi-task learning methods [DeepDR (4), Huang et al. (7)], and foundation-model approaches [UrFound (15), MedViTV2 (58)]. DeepDR requires pixel-level lesion annotations unavailable in EyeQ and is therefore evaluated only on DDR.

#### 3.2.1 Computational efficiency and multi-metric analysis

Tables 6, 7 report computational complexity (parameters, FLOPs, inference time) together with per-class metrics (AUC, sensitivity, specificity, F1, precision) and expected calibration error (ECE) on EyeQ and DDR. With 29.85M parameters and 5.06 GFLOPs, QGDR remains in the same compute envelope as standard single-stream backbones such as ConvNeXt-T (30.15M, 5.02 GFLOPs) and is markedly smaller than the foundation model UrFound (85.80M, 16.86 GFLOPs); inference takes 14.69 ms per image. On EyeQ, QGDR obtains the highest AUC (0.8973) and sensitivity (0.7504) among all compared methods, with a low ECE of 0.0228. On DDR, QGDR is competitive with the best foundation model on AUC (0.9521 vs. 0.9568 for UrFound) and sensitivity (0.8817 vs. 0.8964), and yields a lower ECE (0.0664 vs. 0.0857). Taken together, the results indicate a favorable trade-off between predictive performance, calibration, and computational cost, rather than uniform dominance on every metric.

**Table 6:** Computational complexity and multi-metric performance on EyeQ.

Method	Params (M)	FLOPs (G)	Inference (ms)	AUC	Sens	Spec	F1	Precision	ECE
ResNet-50 (52)	27.92	5.29	12.68	0.8155	0.6432	0.9011	0.6416	0.6400	0.0191
DenseNet-121 (53)	10.38	3.99	19.62	0.8039	0.6435	0.9030	0.6440	0.6446	0.0564
EfficientNet-B4 (54)	18.87	1.92	19.57	0.8098	0.6440	0.8353	0.5734	0.5168	0.1055
Inception-V3 (61)	25.25	4.37	16.38	0.7565	0.5876	0.8897	0.5902	0.5928	0.0771
ConvNeXt-T (50)	30.15	5.02	11.78	0.8505	0.7320	0.8916	0.6879	0.6487	0.0586
CoAtNet-0 (55)	15.53	5.32	14.14	0.8492	0.6933	0.9011	0.6748	0.6572	0.0323
ViT-S (57)	23.93	4.95	18.10	0.8191	0.6435	0.9030	0.6440	0.7563	0.0726
PVTv2-B2 (56)	26.82	4.48	15.65	0.8168	0.7097	0.7683	0.5551	0.4558	0.0815
RegNetY-3.2GF (62)	21.13	3.78	17.50	0.8074	0.6380	0.9105	0.6493	0.6609	0.0563
Huang et al. (7)	23.51	4.13	4.98	0.8819	0.7478	0.8707	0.6735	0.6126	0.0324
UrFound (15)	85.80	16.86	10.03	0.8386	0.6842	0.9344	0.7112	0.7404	0.0183
MedViTV2 (58)	29.58	3.86	10.84	0.8146	0.6744	0.8042	0.5643	0.4851	0.0351
QGDR (Ours)	29.85	5.06	14.69	**0.8973**	**0.7504**	0.8992	0.7082	0.6706	0.0228

AUC, sensitivity, specificity, F1, and precision are computed for the binary referable-DR task (DR grade ≥2); ECE is reported on the 5-class prediction. Best results in bold.

**Table 7:** Computational complexity and multi-metric performance on DDR.

Method	Params (M)	FLOPs (G)	Inference (ms)	AUC	Sens	Spec	F1	Precision	ECE
ResNet-50 (52)	27.92	5.29	12.55	0.8638	0.7562	0.8840	0.7968	0.8419	0.0843
DenseNet-121 (53)	10.38	3.99	19.50	0.8439	0.7686	0.9000	0.8129	0.8625	0.0917
EfficientNet-B4 (54)	18.87	1.92	19.45	0.8805	0.7609	0.8681	0.7916	0.8249	0.1752
Inception-V3 (61)	25.25	4.37	16.41	0.8884	0.7994	0.8424	0.8025	0.8056	0.0646
ConvNeXt-T (50)	30.15	5.02	11.67	0.8290	0.6661	0.9466	0.7157	0.7734	0.0426
CoAtNet-0 (55)	15.53	5.32	14.17	0.8208	0.6337	0.9408	0.6851	0.7454	0.0263
ViT-S (57)	23.93	4.95	17.73	0.8483	0.5916	0.9481	0.6642	0.7572	0.0150
PVTv2-B2 (56)	26.82	4.48	15.62	0.8200	0.7420	0.7419	0.7211	0.7013	0.1241
RegNetY-3.2GF (62)	21.13	3.78	17.60	0.8744	0.7698	0.9545	0.7904	0.8121	0.1378
Huang et al. (7)	23.51	4.13	4.97	0.9040	0.8308	0.9512	0.8789	0.9329	0.1362
DeepDR (4)	108.62	21.28	28.41	0.9314	0.8118	0.9033	0.8412	0.8728	0.1339
UrFound (15)	85.80	16.86	10.43	**0.9568**	**0.8964**	0.8700	0.8722	0.8492	0.0857
MedViTV2 (58)	29.58	3.86	10.74	0.8466	0.6817	0.8565	0.7340	0.7950	0.1598
QGDR (Ours)	29.85	5.06	14.64	0.9521	0.8817	0.8772	0.8678	0.8544	0.0664

AUC, sensitivity, specificity, F1, and precision are computed for the binary referable-DR task (DR grade ≥2); ECE is reported on the 5-class prediction. Best results in bold.

**Backbone generalization analysis** To examine whether the gains stem from the quality-guided design rather than from a particular backbone, we instantiate QGDR with nine backbones spanning CNNs and Transformers (Table 8). Most configurations show consistent improvements over their baselines on both datasets. The accuracy gains are largest for weaker baselines (e.g., Inception-V3 on EyeQ, PVTv2-B2 on DDR), indicating that quality-guided routing partially compensates for architectural limitations. Strong baselines such as ConvNeXt-T show smaller accuracy gains and occasional QWK instability, plausibly because their inductive biases are already well aligned with the task and interact non-trivially with the dynamic routing branch. The overall pattern, namely consistent gains across heterogeneous backbones, supports the interpretation that the benefit comes from explicitly modeling the quality-severity coupling rather than from any single backbone choice.We adopt Swin-T as the default backbone in QGDR because it provides a balanced trade-off across both metrics on both datasets and integrates cleanly with the dynamic routing branch.

**Table 8:** Performance of different backbones with the baseline classifier and with the QGDR framework on EyeQ and DDR.

Backbone	EyeQ				DDR			
	Baseline Acc	QGDR Acc	Baseline QWK	QGDR QWK	Baseline Acc	QGDR Acc	Baseline QWK	QGDR QWK
ResNet-50 (52)	0.6779	0.7532 (+0.0753)	0.5492	0.5706 (+0.0214)	0.7302	0.7380 (+0.0078)	0.7410	0.7306 (−0.0104)
DenseNet-121 (53)	0.6745	0.7517 (+0.0772)	0.5638	0.5774 (+0.0136)	0.7228	0.7579 (+0.0351)	0.7388	0.7454 (+0.0066)
EfficientNet-B4 (54)	0.6392	0.6936 (+0.0544)	0.4802	0.5296 (+0.0494)	0.6597	0.6914 (+0.0317)	0.6683	0.7328 (+0.0645)
Inception-V3 (61)	0.6206	0.7308 (+0.1102)	0.4786	0.5338 (+0.0552)	0.6459	0.7329 (+0.0870)	0.6506	0.7173 (+0.0667)
ConvNeXt-T (50)	0.7551	0.7688 (+0.0137)	0.6527	0.6371 (−0.0156)	0.7662	0.7805 (+0.0143)	0.7555	0.6652 (−0.0903)
CoAtNet-0 (55)	0.7393	0.7560 (+0.0167)	0.6259	0.6311 (+0.0052)	0.6621	0.7675 (+0.1054)	0.7519	0.6326 (−0.1193)
ViT-S (57)	0.6786	0.7135 (+0.0349)	0.6349	0.5334 (−0.1015)	0.6941	0.7570 (+0.0629)	0.7231	0.6079 (−0.1152)
PVTv2-B2 (56)	0.6031	0.6802 (+0.0771)	0.4589	0.5390 (+0.0801)	0.4836	0.6323 (+0.1487)	0.3023	0.5636 (+0.2613)
RegNetY-3.2GF (62)	0.6723	0.7576 (+0.0853)	0.5549	0.5820 (+0.0271)	0.6978	0.7199 (+0.0221)	0.7113	0.7317 (+0.0204)
Swin-T (45)	0.7383	0.7832 (+0.0449)	0.6232	0.6863 (+0.0631)	0.7592	0.8085 (+0.0493)	0.8007	0.8231 (+0.0224)

Δ indicates improvement (+) or degradation (−) relative to the baseline of the same backbone. Blue values indicate improvement over baseline; red values indicate degradation. Numbers in parentheses show the difference (QGDR - Baseline).

**Figure 7:**
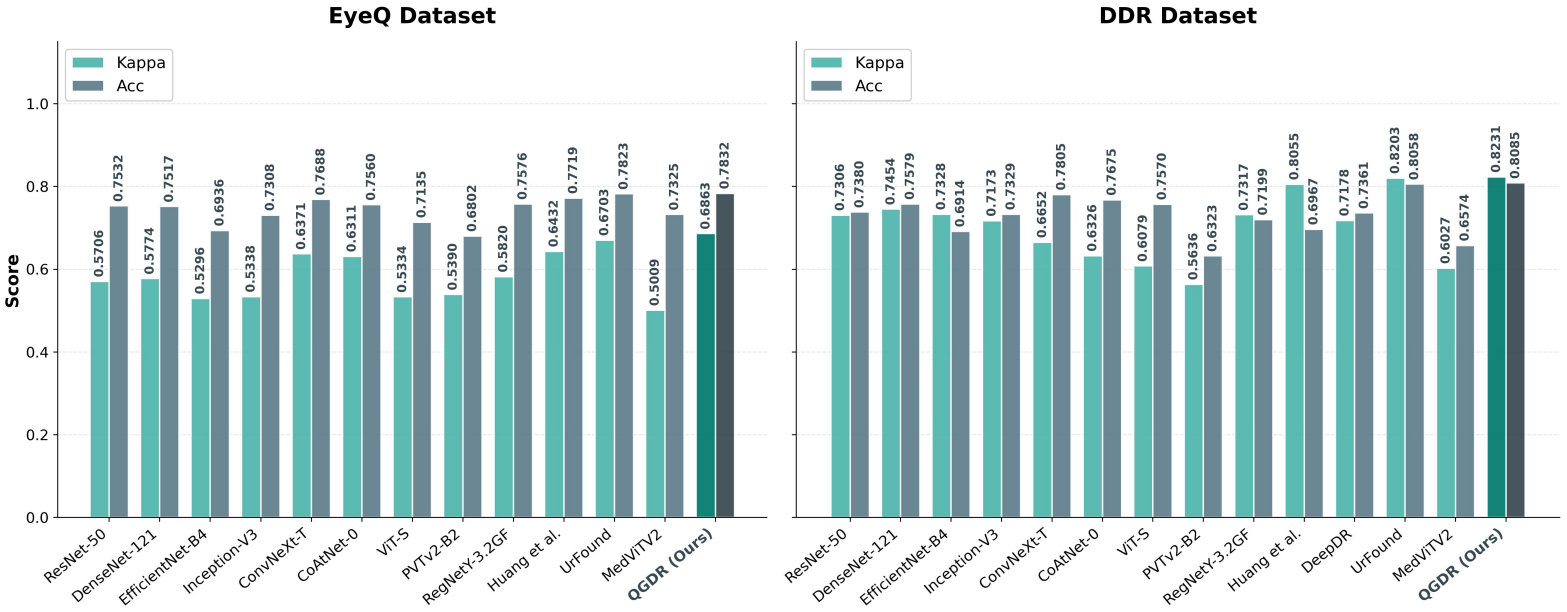
Performance comparison with state-of-the-art methods on EyeQ and DDR.

**State-of-the-art comparison** Table 9 visualized in Figure 7, summarizes the comparison with existing methods. QGDR obtains the best overall accuracy and QWK on both EyeQ (78.32%, 0.6863) and DDR (80.85%, 0.8231). The margin over the foundation model UrFound is small on overall metrics, but it widens on the lowest-quality stratum of both datasets, where QGDR is the highest among all compared methods (77.61% on EyeQ-Reject, 82.21% on DDR-Reject).

**Table 9:** Comparison with state-of-the-art methods for DR grading on EyeQ and DDR.

Method	EyeQ					DDR				
	Acc	QWK	Good	Usable	Reject	Acc	QWK	Good	Usable	Reject
ResNet-50 (52)	0.7532	0.5706	0.7668	0.7497	0.7226	0.7380	0.7306	0.7467	0.7257	0.7069
DenseNet-121 (53)	0.7517	0.5774	0.7645	0.7477	0.7239	0.7579	0.7454	0.7599	0.7776	0.7196
EfficientNet-B4 (54)	0.6936	0.5296	0.7377	0.6810	0.5960	0.6914	0.7328	0.6890	0.6991	0.6940
ConvNeXt-T (50)	0.7688	0.6371	0.7788	0.7711	0.7391	0.7805	0.6652	0.7907	0.7821	0.7512
CoAtNet-0 (55)	0.7560	0.6311	0.7690	0.7578	0.7196	0.7675	0.6326	0.7735	0.7751	0.7410
ViT-S (57)	0.7135	0.5334	0.7219	0.7154	0.6888	0.7570	0.6079	0.7691	0.7635	0.7162
Inception-V3 (61)	0.7308	0.5338	0.7410	0.7315	0.7025	0.7329	0.7173	0.7421	0.7167	0.6667
PVTv2-B2 (56)	0.6802	0.5390	0.7287	0.7097	0.5126	0.6323	0.5636	0.6579	0.5539	0.5717
RegNetY-3.2GF (62)	0.7576	0.5820	0.7638	0.7598	0.7382	0.7199	0.7317	0.7204	0.7435	0.6843
Huang et al. (7)	0.7719	0.6432	0.7824	0.7769	0.7373	0.6967	0.8055	0.7430	0.6785	0.4450
DeepDR (4)	–	–	–	–	–	0.7361	0.7178	0.7504	0.7406	0.6335
UrFound (15)	0.7823	0.6703	**0.7940**	0.7828	0.7509	0.8058	0.8203	0.8061	**0.8042**	0.8070
MedViTV2 (58)	0.7325	0.5009	0.7487	0.7332	0.6891	0.6574	0.6027	0.6710	0.6517	0.5838
QGDR (Ours)	**0.7832**	**0.6863**	0.7857	**0.7837**	**0.7761**	**0.8085**	**0.8231**	**0.8080**	0.8029	**0.8221**

Best results in bold, second-best underlined.

**Figure 8:**
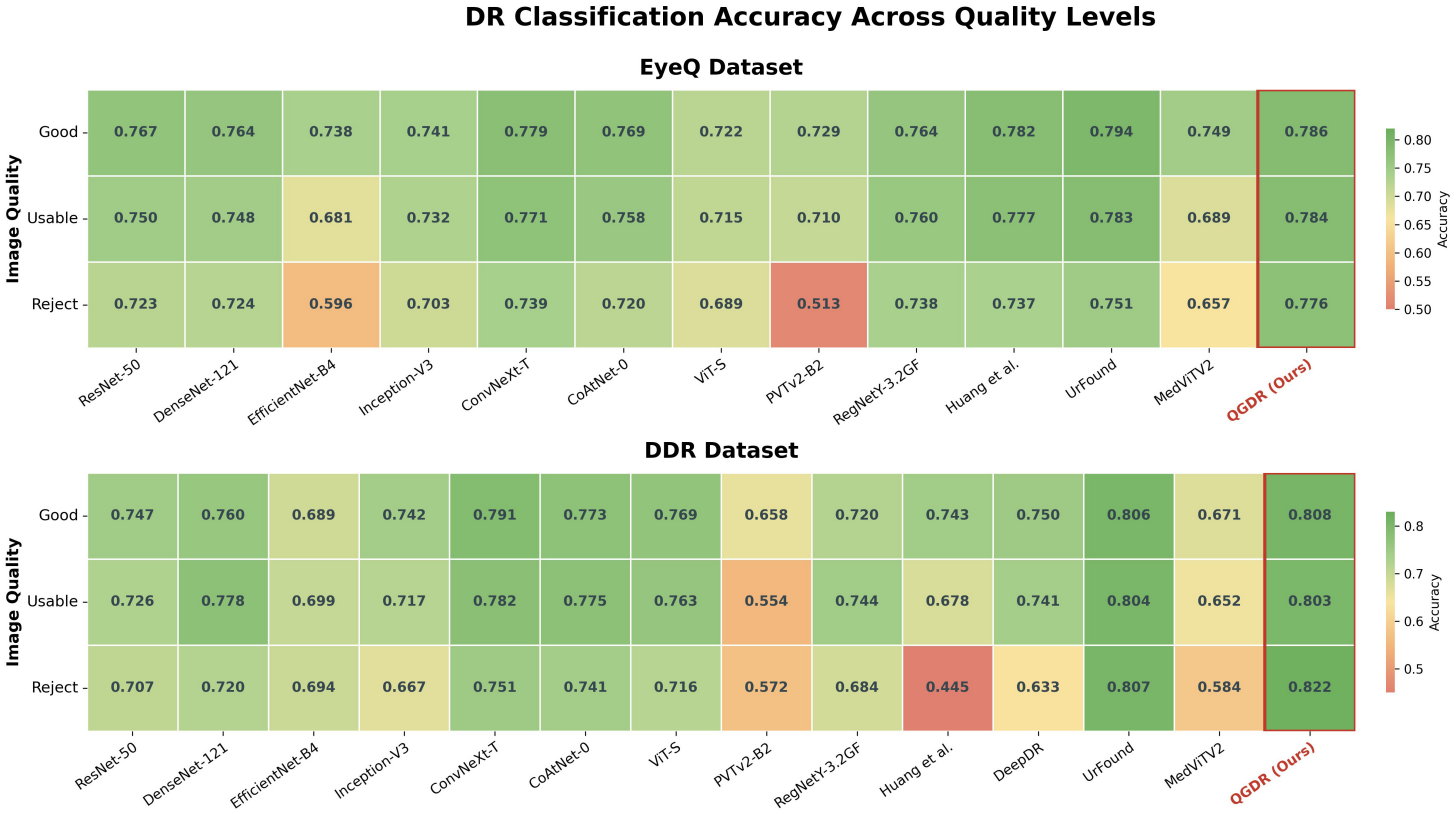
Quality-stratified accuracy on EyeQ **(top)** and DDR **(bottom)**. QGDR maintains accuracy across all quality levels, whereas most baselines degrade from Good to Reject.

The behavior across quality strata is the most distinctive aspect of QGDR (Figure 8). Most baselines lose several accuracy points moving from Good to Reject quality, whereas QGDR shows essentially flat accuracy on EyeQ across the three strata, and on DDR even crosses over to a higher Reject-quality accuracy than Good-quality accuracy (82.21% vs. 80.80%). This pattern is consistent with the quality-severity coupling characterized in Section 1: Reject-quality images on DDR are heavily enriched for advanced DR (Figure 2), and the routing mechanism preferentially assigns these cases to the coarse-scale expert, which is specialized for global pathological patterns. Treating IQA as a continuous conditioning signal therefore allows the framework to retain images that conventional pipelines would either discard or misgrade.

### 3.3 External validation on IDRiD and DeepDRiD

We assess cross-dataset generalization on two external benchmarks: IDRiD (59) (*N* = 516) and DeepDRiD (60) (*N* = 1,600). Models trained on EyeQ or DDR are applied directly to these benchmarks under a test-only protocol. Table 10 reports the results, with the in-domain training source listed in the second column. UrFound (15) and MedViTV2 (58) are evaluated on IDRiD only, because both methods report DeepDRiD as part of their pretraining corpus and a test-only evaluation on DeepDRiD would no longer be out-of-domain. DeepDR (4) is reported only on the DDR dataset, as it involves lesion detection and segmentation tasks for which the EyeQ dataset does not provide ground truth labels.

**Table 10:** External validation on IDRiD and DeepDRiD.

Model	Source	IDRiD (***N*** = 516)			DeepDRiD (***N*** = 1,600)		
		Acc	QWK	AUC	Acc	QWK	AUC
ConvNeXt-T (50)	EyeQ	0.7171	0.8181	0.8978	0.7019	0.7994	0.8952
ConvNeXt-T (50)	DDR	0.7364	0.8504	0.9202	0.7488	0.8267	0.9109
ResNet-50 (52)	EyeQ	0.6531	0.7779	0.8486	0.6906	0.7853	0.8904
ResNet-50 (52)	DDR	0.6957	0.8089	0.9005	0.6987	0.8275	0.8838
ViT-S (57)	EyeQ	0.6124	0.7723	0.7645	0.6369	0.7705	0.8722
ViT-S (57)	DDR	0.6225	0.7768	0.8394	0.6963	0.7860	0.8804
UrFound (15)	EyeQ	0.7326	0.8435	0.9160	–	–	–
UrFound (15)	DDR	**0.7481**	**0.8772**	0.9094	–	–	–
MedViTV2 (58)	EyeQ	0.6981	0.7995	0.8904	–	–	–
MedViTV2 (58)	DDR	0.7132	0.7885	0.9028	–	–	–
DeepDR (4)	DDR	0.7209	0.8519	**0.9313**	0.7288	**0.8401**	**0.9230**
QGDR (Ours)	EyeQ	0.7035	0.8325	0.9047	0.7025	0.8239	0.9090
QGDR (Ours)	DDR	0.7422	0.8556	0.9252	**0.7450**	0.8252	0.9222

Each row reports a single trained model evaluated on external data; “Source” denotes the in-domain training set. Best per-column values in bold.

QGDR transfers consistently to both external cohorts. On IDRiD, the DDR-source QGDR reaches QWK = 0.8556 and AUC = 0.9252, ahead of all generic backbones and competitive with foundation-model approaches such as UrFound (QWK = 0.8772 on its DDR-source variant). On DeepDRiD, the DDR-source QGDR achieves the highest accuracy (0.7450) and a QWK of 0.8252, comparable to the strongest available DR-specific method (DeepDR, QWK = 0.8401). Across both cohorts, the DDR-source models match or outperform their EyeQ-source counterparts. This ordering is expected, as DDR is collected from 147 hospitals across China and its acquisition heterogeneity is closer to that of IDRiD and DeepDRiD than to EyeQ. The relatively higher QWK compared to accuracy reflects that residual errors are predominantly between adjacent DR grades rather than across the severity spectrum, a pattern characteristic of cross-dataset evaluations of ordinal grading models.

### 3.4 Ablation Study

**Figure 9:**
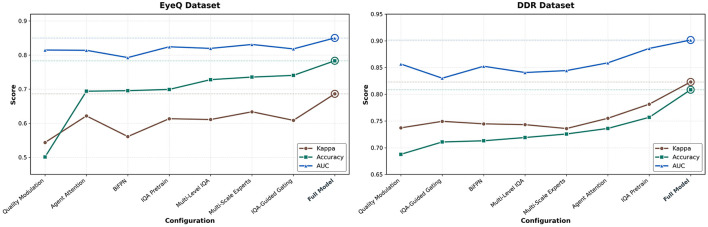
Ablation trends across configurations on EyeQ (**left**) and DDR (**right**). The full model is reached only when all components act jointly.

We conduct ablation experiments on EyeQ and DDR to probe the contribution of each component. Table 1 reports the quantitative results and Figure 9 the corresponding trends.

#### 3.4.1 IQA-DR collaborative learning

The ablation reveals strong interdependence between the IQA and DR components. Adding IQA pretraining alone improves the EyeQ baseline from 69.92% to 72.79%, confirming that quality-aware initialization is beneficial. Adding multi-level IQA features without the corresponding quality modulation, however, drops EyeQ accuracy to 50.11%. Hierarchical quality features therefore inject distractor signal into the spatial branch unless an explicit gating bridge is provided. Once context gating is introduced, accuracy recovers, identifying the gating network as the critical bridge that converts IQA features into useful spatial guidance. The Shuffled-IQA counterfactual on DDR (last row of Table 1) reinforces this point: permuting the pseudo-IQA labels while preserving their marginal distribution drops accuracy from 80.85% to 75.98%, indicating that the IQA pathway exploits the semantic content of the labels rather than acting as a generic auxiliary supervision channel.

#### 3.4.2 Multi-scale feature learning

BiFPN and the multi-scale experts work jointly to address the scale variation of DR lesions. BiFPN supports bidirectional aggregation across resolutions, while the three scale-specialized experts target different lesion sizes: fine-grained microaneurysms at high resolution, intermediate-scale hemorrhages and exudates, and coarse-scale global pathological patterns. Agent attention complements this pipeline by aggregating spatial features through learnable agent tokens with linear complexity. Removing any one of these components consistently reduces accuracy on both datasets, indicating that the multi-scale machinery is best viewed as a joint mechanism rather than as a stack of independent gains.

#### 3.4.3 Quality-adaptive expert routing

**Figure 10:**
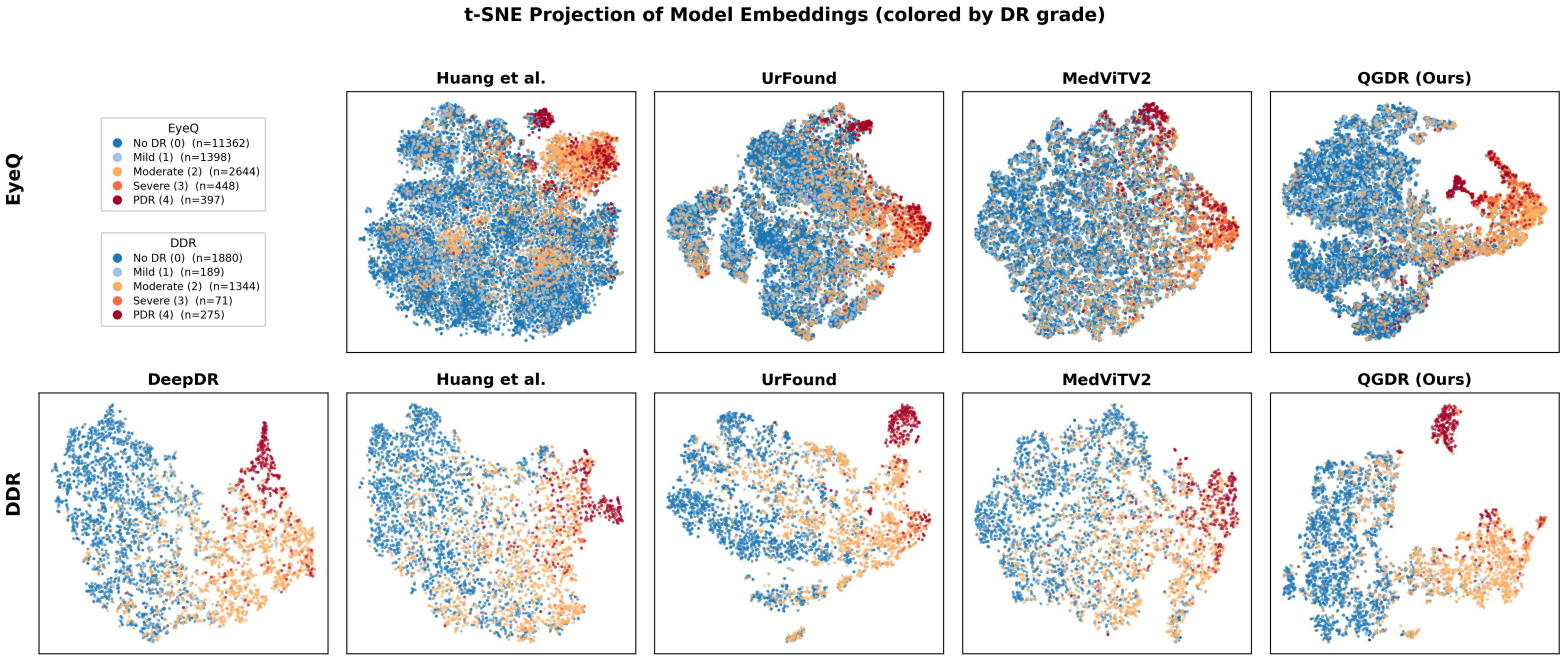
t-SNE visualization of feature embeddings extracted from the EyeQ (top row) and DDR (bottom row) test sets, colored by DR grade. Compared with other models, QGDR tends to show visually cleaner grade-wise separation, and on DDR the PDR cluster appears reasonably well separated from the early grades.

The IQA-guided gating mechanism transforms the predicted quality state into expert routing weights, enabling adaptive expert selection. The full model improves substantially over the strongest single-component variant on both datasets, indicating that the gains arise from the joint operation of IQA pretraining, quality-conditioned attention, multi-scale processing, and quality-adaptive routing rather than from any single mechanism. The t-SNE projections of the final embeddings (Figure 10) provide a complementary view: compared with other models, QGDR yields cleaner grade-wise separation, and on DDR the PDR cluster is more clearly isolated, consistent with the routing of advanced-DR cases to the coarse-scale expert. Together these observations support the view that quality-guided collaborative learning addresses the quality-severity coupling more effectively than treating IQA and DR grading as independent objectives.

### 3.5 Qualitative analysis and visualization

To complement the quantitative results, we examine the per-expert attention patterns and the learned expert routing.

#### 3.5.1 Per-expert attention patterns

**Figure 11:**
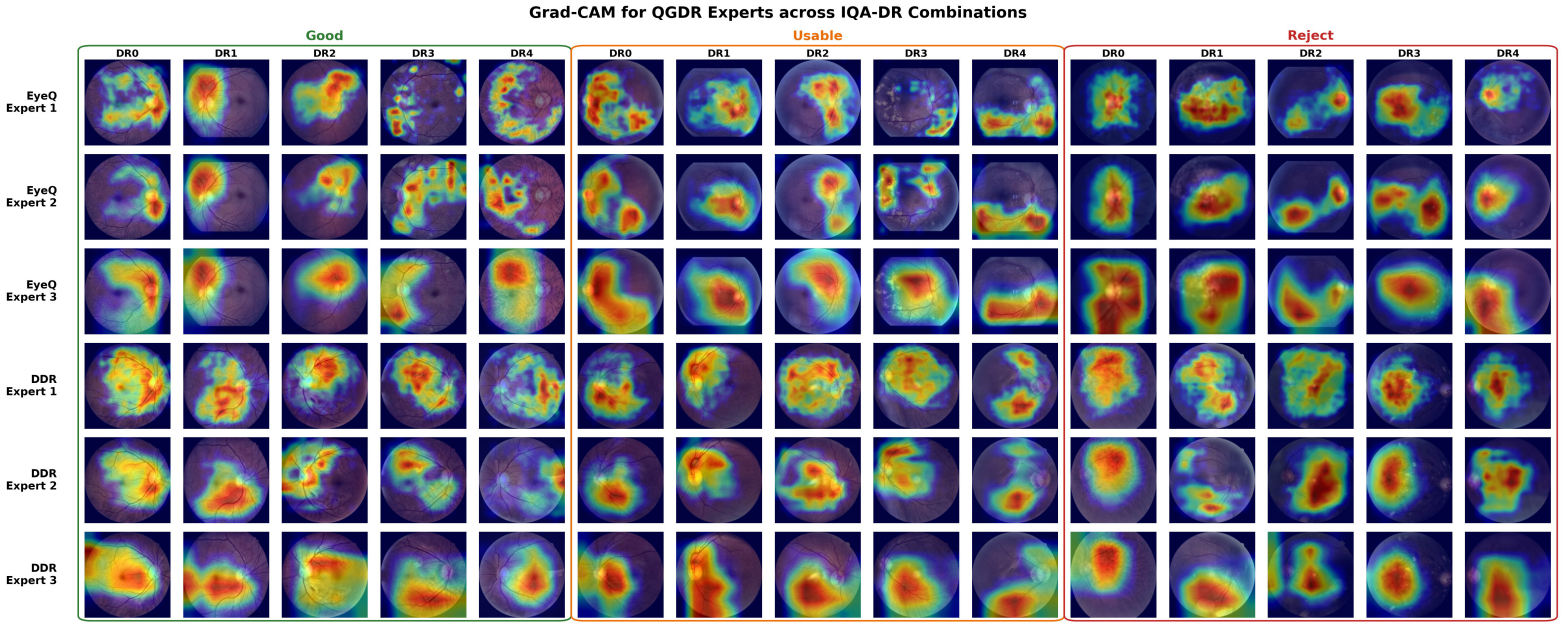
Per-expert Grad-CAM heatmaps on representative samples spanning all IQA-DR combinations on EyeQ (top three rows) and DDR (bottom three rows). The three experts learn distinct attention patterns: Expert 1 (P3, fine scale) localizes dispersed small lesions, Expert 2 (P4, mid scale) emphasizes vascular structures, and Expert 3 (P5, coarse scale) attends to holistic regions.

Figure 11 shows Grad-CAM heatmaps for each of the three experts on samples spanning all nine IQA-DR combinations. The attention maps are clearly differentiated. Expert 1, operating on the fine-scale feature map P3, localizes dispersed small lesions such as microaneurysms and hard exudates. Expert 2, on the mid-scale map P4, places attention on vascular structures and intermediate-size pathology. Expert 3, on the coarse-scale map P5, attends to holistic regions and is the most active on Reject-quality, advanced-DR cases where small lesion cues are unreliable. The fact that the three experts converge on distinct attention semantics, without any pixel-level supervision on lesion location, is consistent with their intended scale specialization in Section 2.3.

#### 3.5.2 Expert routing behavior

**Figure 12:**
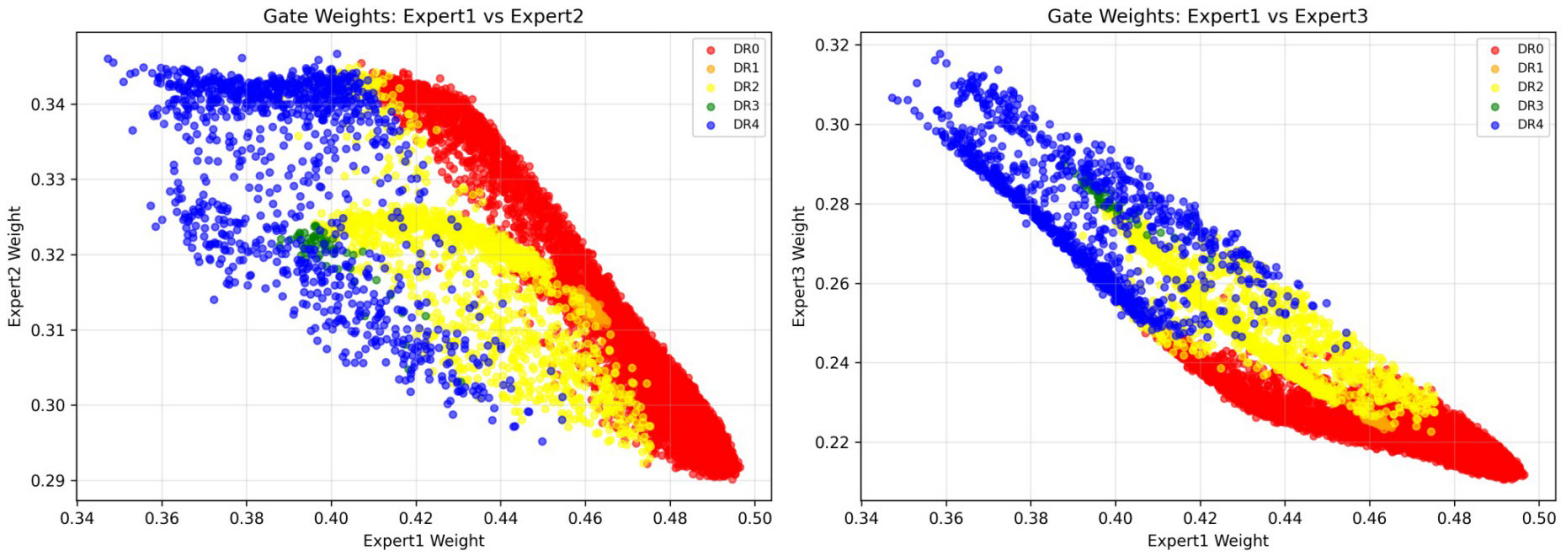
Scatter plot of expert gate weights on the test sets. Each point represents one image, colored by DR grade. DR0 cases concentrate at high Expert 1 weight, while DR4 cases shift toward Expert 2 and Expert 3.

Figure 12 plots the learned gate weights per image, colored by DR grade. DR0 cases concentrate at high Expert 1 weight, moderate cases shift toward Expert 2 dominance, and severe cases lie predominantly on the Expert 3 side. This routing pattern is shaped by two simultaneous signals: the diversity loss (Equation 8) provides soft DR-grade-specific target weights, and the quality-to-expert bias term **b**_IQA_ in the gating network (Equation 6) shifts the routing as a function of the predicted quality state. Because Reject-quality images are enriched for advanced DR (Section 1), the two signals act in the same direction, and the routing learns a coupling between image quality, lesion scale, and DR grade that is consistent with clinical expectation. Importantly, this coupling emerges through soft supervision rather than through hard constraints, leaving the model free to deviate from the prior on individual cases when the image-level evidence requires it.

## 4 Discussion

### 4.1 From quality filtering to quality guidance

The central contribution of QGDR is conceptual rather than purely architectural. We re-frame image quality from a binary gating decision into a continuous guidance signal that conditions feature learning and expert routing throughout the diagnostic pipeline. This re-framing is motivated by an underappreciated phenomenon in real-world DR screening, the quality-severity coupling. Our dataset-level analysis on EyeQ and DDR (Figure 2) shows that referable DR cases are systematically enriched in low-quality images, with an odds ratio of 4.17 on DDR (ρ = 0.420, *p* < 0.001). Low-quality images are therefore not screening failures to be discarded, but precisely the population where automated assistance is most consequential.

Existing paradigms do not engage with this coupling. Enhancement methods modify inputs before diagnosis and may distort the small lesions they attempt to reveal. Quality-filtering pipelines exclude exactly the cases that most need diagnostic support. Multi-task IQA-DR formulations share representations between the two tasks, but treat them as parallel objectives and do not let quality predictions actively steer disease-specific feature processing. QGDR closes this gap by making quality an explicit conditioning variable for spatial attention and expert selection, turning a source of error in conventional pipelines into a usable signal.

### 4.2 Behavior on degraded images

A noteworthy aspect of the empirical results is the behavior on the lowest-quality stratum. On DDR, accuracy on Reject-quality images (82.21%) exceeds accuracy on Good-quality images (80.80%); on EyeQ, the gap between Good (78.57%) and Reject (77.61%) is essentially closed. The quality-stratified analysis (Figure 8) shows that nearly all competing methods, including the foundation model UrFound, exhibit monotonic degradation from Good to Reject quality, so this flattening is not an artifact of dataset composition.

The mechanism is visible in the routing analysis (Figure 12). Two complementary signals drive expert selection in QGDR: the diversity loss provides DR-grade-specific soft targets, and the quality-to-expert bias term in the gating network shifts routing as a function of the predicted quality state. Because Reject-quality images on DDR are heavily enriched for advanced DR, the two signals act in the same direction, and the gate consistently shifts toward Expert 3, the coarse-scale specialist for global pathological patterns. The framework therefore exploits, rather than resists, the quality-severity coupling that conventional pipelines treat as noise.

### 4.3 Methodological insights from the ablation

The ablation study (Table 1) yields a design principle worth highlighting: hierarchical quality features alone are not enough. Adding the multi-level IQA module without the quality-conditioned context gating drops EyeQ accuracy from 72.79% to 50.11%, a 23-point reduction. The additional features inject distractor signal into the spatial branch unless they are routed through an explicit gating bridge, and once context gating is reintroduced, accuracy recovers. This pattern is consistent with prior observations in multi-task learning: auxiliary signals require explicit integration, not merely parallel supervision. In our setting, IQA must function as a conditioning variable, not as a side objective.

The Shuffled-IQA counterfactual on DDR sharpens this conclusion. Permuting the pseudo-IQA labels while preserving their marginal distribution drops DR accuracy from 80.85% to 75.98%, indicating that QGDR exploits the semantic content of the labels rather than benefiting from a generic auxiliary supervision channel. Cross-architecture instantiation across nine backbones (Table 8) further shows that the gain is largely backbone-agnostic: improvements are largest on weaker baselines and modest on strong ones, with occasional QWK instability when adaptive routing interacts with strongly biased backbones such as ConvNeXt-T.

### 4.4 External generalization and clinical implications

The external evaluation on IDRiD and DeepDRiD (Table 10) tests whether QGDR generalizes beyond the in-domain distribution under a test-only protocol. The DDR-source models consistently match or outperform their EyeQ-source counterparts on both external cohorts, an ordering that aligns with the acquisition heterogeneity of the training source: DDR is collected from 147 hospitals across China and is closer in distribution to IDRiD and DeepDRiD than EyeQ, which is dominated by a single screening program. This pattern indicates that QGDR's quality-conditioned routing transfers without re-tuning when the source training distribution itself spans diverse clinical conditions.

From a clinical perspective, current screening programs face a coverage-reliability dilemma. Strict quality filtering ensures reliable diagnoses but reduces screening coverage, particularly in underserved populations and resource-limited settings where imaging conditions are suboptimal; accepting all images uniformly risks misdiagnosis. QGDR offers an intermediate operating point: degraded images can be retained in the pipeline with calibrated diagnostic confidence (ECE = 0.0228 on EyeQ, 0.0664 on DDR) rather than triaged out and resubmitted. The expert routing also provides a coarse form of interpretability, since clinicians inspecting an output can observe which scale-specialized expert dominated, gaining a partial answer to whether the prediction relied on fine-scale lesion detection or on coarse-scale global patterns.

### 4.5 Limitations and future directions

Several limitations temper the present claims and motivate follow-up work. First, although QGDR couples three modules, the resulting model (29.85M parameters, 5.06 GFLOPs) sits within the compute envelope of standard backbones such as ConvNeXt-T (30.15M, 5.02 GFLOPs) and is substantially smaller than foundation models such as UrFound (85.80M, 16.86 GFLOPs). Nevertheless, the multi-module design reduces interpretability relative to a single-stream classifier. Future work could integrate *post-hoc* explanation tools tailored to mixture-of-experts routing, or distill QGDR into a single-stream student model for deployment.

Second, the framework relies on quality annotations or pseudo-labels for IQA supervision. Quality labels are subjective and can be inconsistent across datasets, and the pseudo-label validation in Section 3.1 only partially addresses this concern. Self-supervised quality estimation, for instance via contrastive pretext tasks on degraded–restored image pairs, could remove the annotation dependency.

Third, although we evaluate on EyeQ, DDR, IDRiD, and DeepDRiD, all four are research-curated benchmarks. True clinical validation requires prospective evaluation on consecutively collected screening data with diverse imaging devices, varying operator skill, and direct comparison against ophthalmologist grading. We regard this as the most important next step.

Fourth, the IQA-DR coupling exploited by QGDR is supported empirically through the Shuffled-IQA experiment, the cross-architecture consistency, and the quality-stratified accuracy results, but is not formally identified as a causal mechanism. The relationship is plausibly anticausal, in the sense that disease severity causally influences imaging fidelity (advanced pathology degrades the optical path) and the predictor learns to invert this generative direction. Recent developments in causal and anticausal representation learning, and in invariant prediction across heterogeneous environments, offer principled tools for distinguishing genuine task coupling from environment-specific spurious correlation. Embedding QGDR-style quality conditioning within such frameworks (treating, for instance, acquisition site or imaging device as the environment variable) would allow stronger generalization guarantees than empirical cross-dataset transfer can provide.

Finally, the present study addresses DR only. The quality-severity coupling phenomenon plausibly recurs in other ophthalmic conditions in which the pathology itself degrades image acquisition: glaucoma (media opacities co-occurring with disc cupping), age-related macular degeneration (geographic atrophy degrading macular reflectance), and retinal vein occlusion (hemorrhage obscuring vasculature). Extending QGDR-style quality conditioning to multi-disease and multi-modality settings is a natural direction.

## 5 Conclusion

This paper addressed a fundamental tension in automated DR screening: the diagnostically most critical images are systematically the lowest in quality, because advanced disease itself degrades fundus imagery. Conventional pipelines that filter or uniformly process such images implicitly discard or mishandle the patients most in need of intervention. Resolving this tension requires re-framing image quality assessment from a binary preprocessing decision into a continuous guidance signal that actively conditions feature learning and expert routing.

To realize this paradigm, we proposed QGDR, a quality-guided dynamic routing framework that integrates three coordinated components: a multi-level IQA module that extracts hierarchical quality features across backbone stages; a quality-conditioned context gating mechanism that modulates spatial attention according to the predicted quality state; and an adaptive gated fusion mechanism that routes inputs to scale-specialized experts, with high-quality images preferentially activating fine-scale experts for subtle lesions and degraded images relying on coarse-scale experts for global pattern recognition.

Empirically, QGDR achieves competitive performance on EyeQ (78.32% accuracy, 0.6863 QWK) and DDR (80.85% accuracy, 0.8231 QWK) against representative CNN, transformer, multi-task, and foundation-model baselines, while remaining within the compute envelope of standard single-stream backbones. The most distinctive empirical signature is preserved accuracy across quality strata: on DDR, Reject-quality accuracy (82.21%) exceeds Good-quality accuracy (80.80%), reflecting the coupling between low image quality and advanced disease. Test-only evaluation on IDRiD and DeepDRiD indicates that the framework generalizes beyond the in-domain distribution.

Two methodological observations support the overall argument. The dataset-level coupling analysis (Spearman ρ = 0.420, OR = 4.17 on DDR, *p* < 0.001) provides a quantitative grounding for treating low-quality images as a distinct, severity-enriched population rather than as preprocessing failures. The ablation indicates that hierarchical quality features must be paired with explicit context gating, a design principle likely transferable to other settings in which auxiliary clinical signals must be integrated into perception models. The Shuffled-IQA counterfactual confirms that QGDR uses the semantic content of the IQA labels rather than benefiting from a generic auxiliary supervision channel.

Beyond the technical contribution, QGDR offers a candidate solution to the coverage-reliability dilemma faced by current screening programs. Rather than triaging degraded images out of the pipeline, they can be retained with calibrated diagnostic confidence, broadening screening access in resource-limited settings without sacrificing accuracy. The expert routing provides a coarse interpretability signal indicating whether a prediction relied on fine-scale lesion detection or on coarse-scale global patterns.

Important next steps include prospective clinical validation against ophthalmologist grading on consecutively collected screening data, self-supervised quality estimation to remove the annotation dependency, *post-hoc* interpretability tools tailored to expert-routed predictions, and extension to other ophthalmic conditions in which the quality-severity coupling plausibly recurs. More broadly, the present results suggest that degraded inputs in medical imaging may be more usefully treated as informative signals than as preprocessing failures, provided that architectural support is in place to condition on them rather than around them.

## Data Availability

Publicly available datasets were analyzed in this study. This data can be found here: The EyeQ dataset used in this study is publicly available at https://github.com/HzFu/EyeQ. The DDR dataset can be accessed at https://github.com/nkicsl/DDR-dataset. The FIQS dataset is available at https://figshare.com/articles/dataset/FIQS_Dataset_Fundus_Image_Quality_Scores_/28129847.
